# The antioxidative effects of acidic-, alkalic-, and enzymatic-extractable mycelium zinc polysaccharides by *Pleurotus djamor* on liver and kidney of streptozocin-induced diabetic mice

**DOI:** 10.1186/s12906-015-0964-1

**Published:** 2015-12-18

**Authors:** Jianjun Zhang, Guangyuan Meng, Chen Zhang, Lin Lin, Nuo Xu, Min Liu, Fangyuan Cui, Le Jia

**Affiliations:** College of Life Science, Shandong Agricultural University, Taian, 271018 PR China; The Central Hospital of Taian, Taian, Shandong 271000 PR China

**Keywords:** *Pleurotus djamor*, Oxidative stress, Diabetes, Mycelium zinc polysaccharides

## Abstract

**Background:**

Edible mushrooms, especially the genus of *Pleurotus*, have been well studied for their nutrition as well as non-toxic medicinal properties. Recently, much attention has been paid to the therapeutic values of mushrooms in genus of *Pleurotus* with diabetes mellitus (DM), which was a complex metabolic disorder that induced by increased oxidative stress and characterized by hyperglycemia. However, scare attention has been paid to polysaccharides from *P. djamor*. Meanwhile, zinc is an essential trace element in the human body and it participates in various pathways of metabolism. Therefore, the objective of present study was aimed to evaluate the protective effects of the three extractable mycelium zinc polysaccharides (MZPS), including acidic-MZPS (Ac-MZPS), alkalic-MZPS (Al-MZPS) and enzymatic-MZPS (En-MZPS), on the liver and kidneys in diabetic mice induced by streptozocin (STZ) aiming to better understand the possible hypoglycemic mechanisms and their health benefits.

**Methods:**

The Ac-, Al-, and En-MZPS were extracted with hydrochloric acid (1 M), sodium hydroxide (1 M) and snailase (4 %) from *P. djamor* zinc-enriched mycelium, respectively. The diabetic mice were induced by injection of STZ. Besides the histopathological analyses of liver and kidney, the following biochemical analysis were processed to investigate the antioxidative effects, including activities of superoxide dismutase (SOD), GSH peroxide (GSH-Px) and catalase (CAT), and contents of malondialdehyde (MDA) in liver and kidney homogenate; activities of alamine aminotransferase (ALT) and aspertate aminotransferase (AST), and levels of urea nitrogen (BUN), creatinine (CRE), total cholesterol (TC), albumin (ALB), high-density lipoprotein cholesterol (HDL-C), low-density lipoprotein choles-terol (LDL-C) and very low-density lipoprotein cholesterol (VLDL-C) in serum.

**Results:**

Results showed that the activities of SOD, GSH-Px and CAT were significantly increased, the MDA contents remarkably reduced, and the values of ALT, AST, BUN, CRE, TC, LDL-C and HDL-C observably mitigated in the liver, kidneys and serum of diabetic mice by these three polysaccharides treatment. Biochemical and histopathological analyses also showed that MZPS could alleviate liver and kidneys injury.

**Conclusion:**

These results demonstrated that Ac-, Al-, and En-MZPS possessed potent antioxidant activities, and could be used as a potentially functional food for the prevention of diabetes and its complications induced by STZ.

## Background

Nowadays, mushrooms have become attractive not only due to their excellent taste, but also thanks to their healthy properties [1]. Mushrooms have been traditionally used in medicine as a source of new drugs due to the occurrence of a wide number of bioactive compounds involved in the prevention and treatment of several diseases, such as cancer, inflammatory disease, heperlipidemia, diabetes, and liver damage [2]. Among the most important metabolites obtained from mushrooms, polysaccharides have gained much attention owing to their demonstrated bioactivities, such as ability of scavenging the free radicals, anti-tumor [3], immunoregulation [4], antioxidant [5], anti-aging [6], anti-diabetic [7] and hepatoprotective effects [8]. In addition, a concerted effort to improve the nutrition with polysaccharides supplementation has been ongoing in the bioavailability areas.

In recent years, increasing evidence in both experimental and clinical studies have pointed out that increased oxidative stress, accompanied by increased production of reactive oxygen species (ROS) or impaired antioxidant defenses, is a widely accepted participant in the development, progression and pathogenesis of diabetes and its complications, such as diabetes mellitus (DM) [9–13]. DM is a complex metabolic disorder characterized by hyperglycemia due to the improper utilization of glucose [14]. According to the statistics from the International Diabetes Federation (IDF, 2012), more than 371 million people are diagnosed to have diabetes and the number of people with DM are rising exponentially. Undoubtedly, the DM has been regarded as one of the greatest medical and socioeconomic challenges worldwide [15]. However, mechanisms by which increased oxidative stress is involved in the diabetic complications are not well defined. Many factors including viral infection, autoimmune disease, unnormal diet and environmental factors have potential in raising the risk of DM. Streptozotocin (STZ), an antibiotic produced by *Streptomyces achromogenes*, is frequently used to induce DM in experimental animals due to its toxic effects on generation of ROS causing oxidative damage on the liver and kidney for evaluating the therapeutic potential of antidiabetics [16]. Evidence is accumulating which indicates that the hpyerglycemic-related activities of polysaccharides may be linked to their known antioxidant and pre-oxidant properties, suggesting that mushroom polysaccharides have promising activity for the prevention of diabetes. Therefore, there is a strong need for safe and effective oral anti-hyperglycaemic agents that provide an alternative option for preventing, and treating diabetes and its complications.

It has been reported that polysaccharides isolated from the genus of *Pleurotus* showed many biological activities. Documents has been reported that some species in the genus of *Pleurotus*, such as *P. eryngii* [17], *P. ostreatus* [18], *P. Tuber-regium* [19], and *P. cystidiosus* [20] etc., showed potential effects in treatment with DM. In this regard, the polysaccharides extracted from *P. djamor*, a new species classifies in the genus of *Pleurotus* that appreciated as an edible and medicinal mushrooms in China, may play a promising role in the treatment with liver and kidney damage that induced by hyperglycemia. However, to our best knowledge, so far there is scare publication information about the antioxidant effects of polysaccharides extract from mycelium of *P. djamor*, and there are no available studies regarding the role of ROS in STZ-induced liver and kidney damage. Meanwhile, zinc is an essential trace element in the human body and it participates in various pathways of metabolism [21]. Interestingly, it has been proved by our previous report that, after submerged fermentation with zinc-compound (zinc acetate), zinc-enriched polysaccharides is shown to exhibit significantly higher bioactivities than regular polysaccharides (The work of enrichment of zinc and *P. djamor* has been processed, but the data were not shown here) [22]. Urgently, it is quite necessary and significative to explore polysaccharides from the zinc-enriched mycelia of *P. djamor* and evaluate their protective effects against DM.

The objective of present study was conducted to evaluate the protective effects of the three extractable mycelium zinc polysaccharides (MZPS), including acidic-MZPS (Ac-MZPS), alkalic-MZPS (Al-MZPS) and enzymatic-MZPS (En-MZPS), on the liver and kidneys in diabetic mice induced by STZ-injection in order to better understand the possible hypoglycemic mechanisms and their health benefits.

## Methods

### Organism and chemicals

The strain of *P. djamor* was provided and identified by Department of Microbiology of Shandong Agricultural University (Taian, Shandong), and the deposition number was SAB-01. The diagnostic kits of superoxide dismutase (SOD), glutathione peroxidase (GSH-Px), catalase (CAT), and malondialdehyde (MDA) were purchased from Nanjing Jiancheng Bioengineering Institute (Nanjing, China). Snailase and streptozocin (STZ) was purchased from Sigma Chemicals Company (St. Louis, USA). All other chemicals were of analytical grade and purchased from local chemical suppliers.

### Preparation of Ac-, Al-, and En-MZPS

The liquid fermentation technology was used to produce MZPS by *P. djamor*. Each 250-mL flask, containing 100 mL basal medium (g/L) of potato 200, glucose 20, KH_2_PO_4_ 1.5, MgSO_4_ · 7H_2_O 1 and with ZnSO_4_ (3 g/L, 3 mL) for supplying the zinc, was kept cultivation at 25 °C with a shaking of 140 rpm for 10 days. After filtration, concentration, sterilization and lyophilization, the mycelium was extracted with proper volumes of hydrochloric acid (1 M), sodium hydroxide (1 M) and snailase (4 %) at 35 °C for 4 h (1:4, w/v). The supernatant liquids were precipitated by ethanol precipitation (1:3, v/v) at 4 °C overnight, collected by centrifugation and deproteinated by employing the Sevage method [23], respectively. Finally, the deproteinated precipitates were pooled and lyophilized to yield Ac-, Al-, and En-MZPS, which were used for further studies.

### Animal experiments

The experiments were performed as approved by the institutional animal care and use committee of Shandong Agricultural University, and in accordance with the Animals (Scientific Procedures) Act. 1986 (amended 2013). Kunming strain mice, weighed 20 ± 2 g (4 weeks old), was purchased from Taibang Biological Products Ltd. Co. (Taian, China). The mice were acclimated for 2 days prior to dosing, during which time they had free access to food and water *ad libitum* (temperature 22 ± 1 °C, humidity 60 to 65 %, lights on 12 h every day). For diabetes induction, mice were fasted overnight and then intraperitoneally injected with 120 mg/Kg STZ (freshly dissolved in sodium citrate buffer (pH 4.5) and immediately injected within 20 min of preparation) [24–26]. Control mice (NC groups) were intraperitoneally injected with citrate buffer alone. Two days after STZ injection, the diabetic state was assessed by measuring blood glucose (GLU) levels after fasting overnight. Mice with GLU levels over 11 mM were considered as successful models of diabetic. All diabetic mice were randomly divided into 11 groups including model groups (MC), positive groups (PC), nine dosage groups of three high dosage groups (800 mg/Kg), three middle dosage groups (400 mg/Kg), and three low dosage groups (200 mg/Kg). During the gavage procedure, the NC and MC groups received vehicleonly (distilled water) only, while the dose groups received Ac-, Al-, and En-MZPS at dosage of 800, 400, 200 mg/Kg, respectively. The vehicle or test drugs were suspended in distilled water. The treatment with polysaccharides was started 5 days after STZ injection and was lasted for 2 weeks. At the end of the experimental, the mice were fasted overnight, and then sacrificed by exsanguinations under diethyl ether anesthesia.

Serum was obtained from the blood by centrifugation (14,000 × g, 4 °C). Alamine aminotransferase (ALT) activity, aspertate aminotransferase (AST) activity, urea nitrogen (BUN) levels, creatinine (CRE) levels, total cholesterol (TC) levels, albumin (ALB) levels, high-density lipoprotein cholesterol (HDL-C) levels, low-density lipoprotein choles-terol (LDL-C) levels and very low-density lipoprotein cholesterol (VLDL-C) levels of serum were measured using automatic biochemical analyzer (ACE, USA).

The liver and kidney index was calculated by (liver weight)/(body weight) (g/100 g). The atherogenic index (AI) was calculated as (TC–HDL-C)/HDL-C [27].

The liver and kidney were rapidly removed, weighed and homogenized (1:9, w/v) immediately in phosphate buffer solutions (PBS, 0.2 M, pH 7.4). After centrifugation (5000 × g, 4 °C) for 20 min, the supernatants were collected for further biochemical analysis. The activities of SOD, GSH-Px and CAT, and contents of MDA in liver and kidney homogenates were determined by the commercial reagent kits according to the instructions.

Liver and kidney cortex samples were immersed in the PBS buffer containing 10 % formalin (pH 7.4) for over 24 h and embedded in paraffin. Thin sections (4–5 μm thickness) were prepared by using a microtome and stained with hematoxylin-eosin. Each section was photographed under microscope to show the histopathological changes (×400 magnifications).

### Acute toxicity

Acute toxicity test in mice was performed according to the method of Chao et al. [28]. Male mice were divided into three dose-groups and one control group (Ten mice in each group). The dose-groups was performed by using oral dosages of Ac-, Al-, and En-MZPS (5000 mg/kg). Control group received the isometric saline solutions. All mice were allowed for food *ad libitum* for 14 days and kept under regular observation for any mortality or behavioral changes (irritation, restlessness, respiratory distress, abnormal locomotion, and catalepsy etc.).

### Statistical analysis

All data were presented as the mean ± standard deviation (SD) from three independent experiments. Significant differences among the groups were determined by one-way ANOVA using the IBM SPSS Statistical software package program. *P* < 0.05 was considered to be statistically significant.

## Results

### Body weight, blood glucose levels, liver index and kidney index

**Fig. 1:**
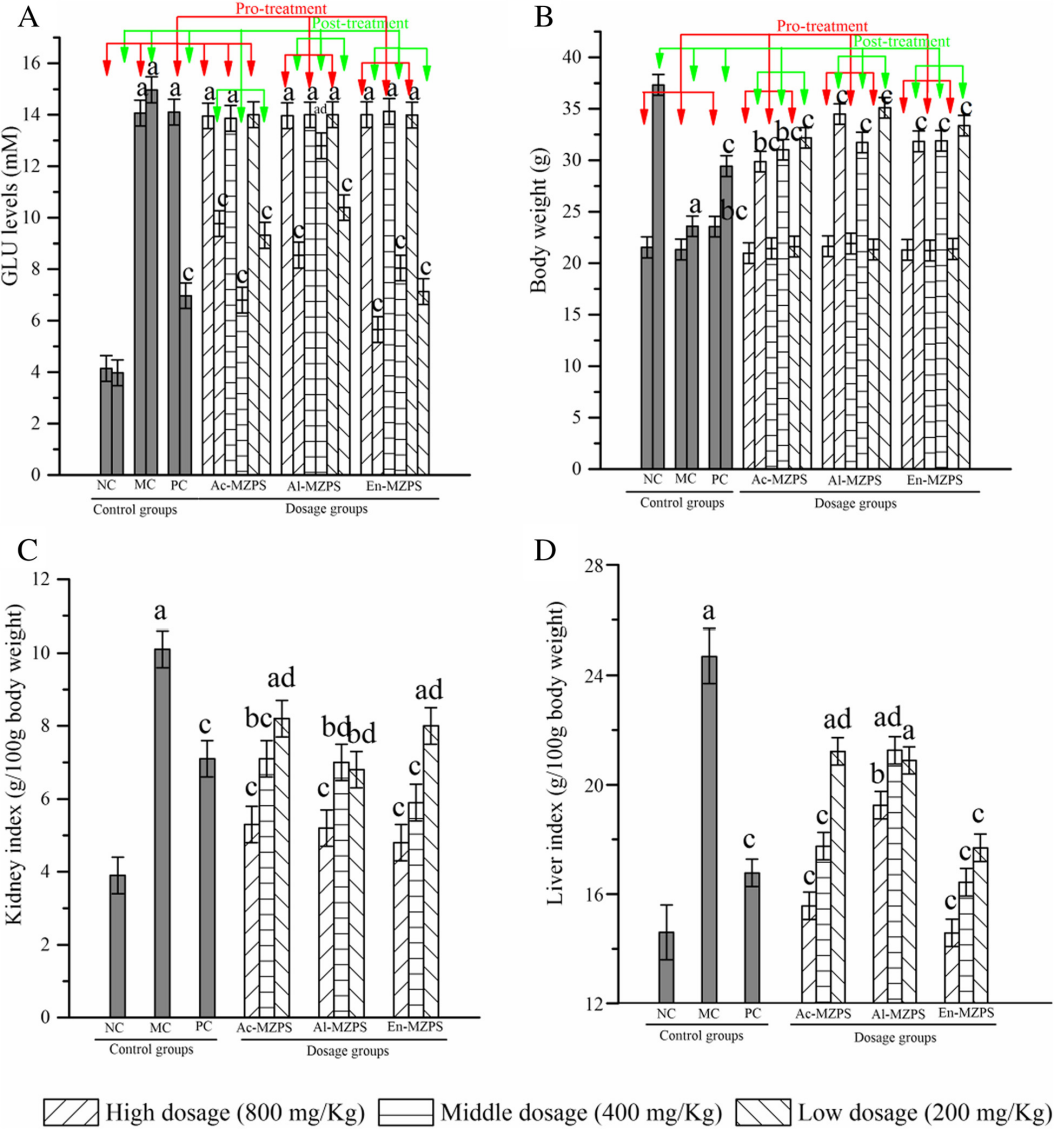
Effects of Ac-, Al-, and En-MZPS on (**A**) GLU levels, (**B**) Body weight, (**C**) Liver index, and (**D**) Kidney index in STZ-treated mice. The values are reported as the mean ± SD of ten mice per group: (a) *P* < 0.01 and (b) *P* < 0.05 compared with NC groups; (c) *P* < 0.01 and (d) *P* < 0.05 compared with the MC groups

The effects of Ac-, Al-, and En-MZPS on GLU levels, body weight, liver index, and kidney index of STZ-induced diabetic mice were presented in Fig. 1.

The diabetic mice exhibited a significant increase in GLU levels as compared to the NC group (Fig. 1A, *P* < 0.01). The administration of Ac-, Al-, and En-MZPS at different doses caused significant decrease in GLU levels in diabetic mice when compared with untreated diabetic mice (MC group, *P* < 0.01 or *P* < 0.05). Detailedly, compared with the MC group, at the end of the experiment, the GLU levels of mice in Ac-, Al-, and En-MZPS at three doses (800, 400, and 200 mg/Kg) groups were reduced by 34.74 ± 7.57, 57.58 ± 3.15, 37.74 ± 6.36, 42.89 ± 2.77, 14.50 ± 4.51, 30.60 ± 4.12, 62.26 ± 6.21, 46.23 ± 8.32, and 52.37 ± 5.07 %, respectively.

Simultaneously, the body weight of diabetic mice was also observed. As shown in Fig. 1B, the body weight of mice in NC group increased regularly during the administration. However, the diabetic mice in MC group expressed a significant loss of body weight as compared to NC group (*P* < 0.01). Compared with the MC group, at the end of the experiment, the body weight of mice in Ac-, Al-, and En-MZPS at three doses (800, 400, and 200 mg/Kg) groups were increased by 26.68 ± 4.17, 31.59 ± 5.22, 36.51 ± 3.41, 33.59 ± 1.97, 34.56 ± 1.82, 48.85 ± 3.22, 35.07 ± 6.06, 35.28 ± 3.27, and 41.56 ± 2.68 %, respectively (all *P* < 0.01).

The kidney and liver indexes of mice in all groups were shown in Figs. 1C and D. Apparently, both significant increases could be seen in MC group when compared to the NC group (*P* < 0.01) in liver and kidney. However, the increases in liver and kidney could be mitigated by pretreatment with Ac-, Al-, and En-MZPS at three different doses (*P* < 0.05 or *P* < 0.01), respectively.

Meanwhile, when tested at a dosage of 400 mg/Kg, glibenclamide as a standard antioxidant (hyperglycemic agent) also effectively protected liver and kidney against the oxidative damage induced by STZ (Fig. 1).

### Effects of Ac-, Al-, and En-MZPS on SOD, GSH-Px, CAT, and MDA

**Fig. 2:**
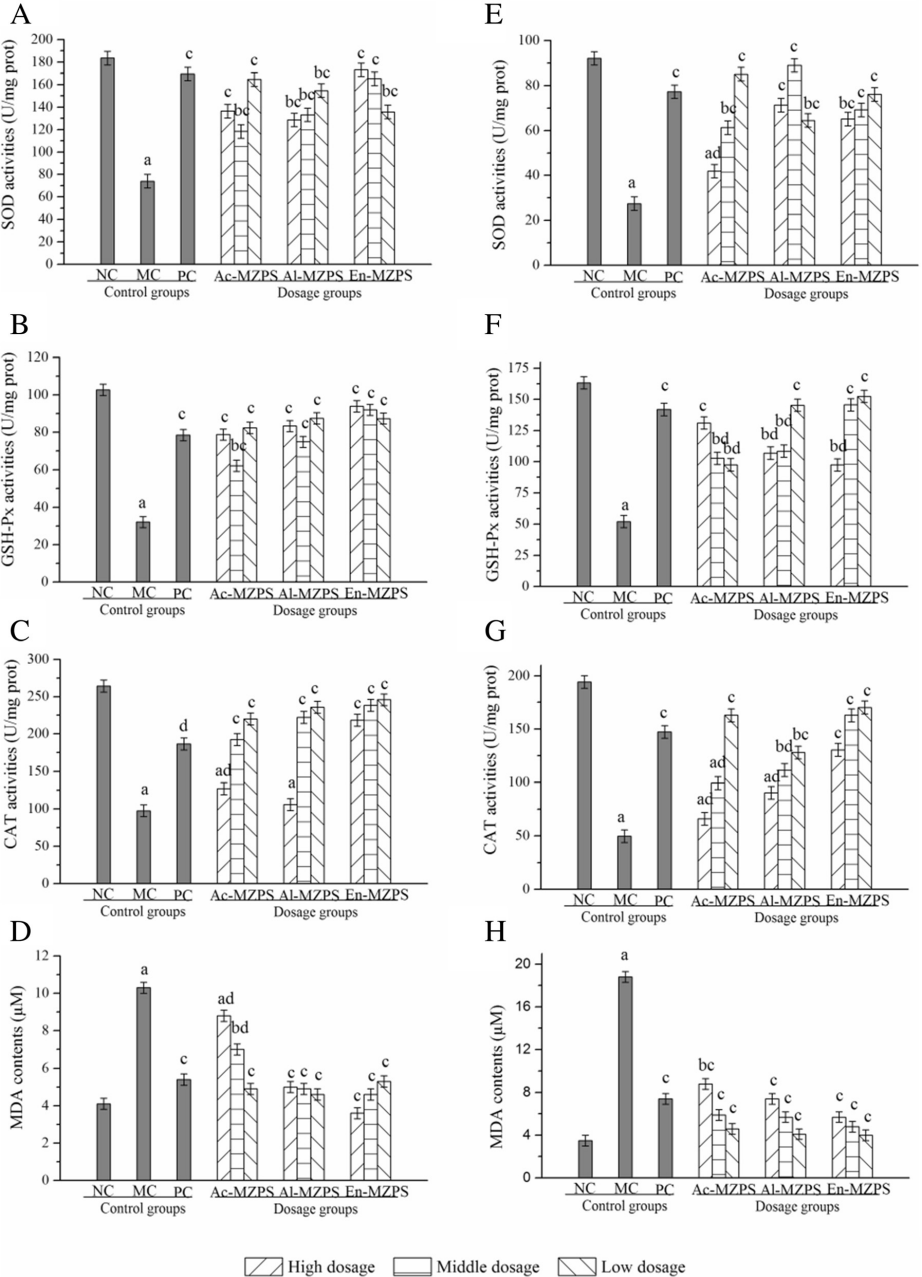
Effects of Ac-, Al, and En-MZPS on the activities of (**A**) SOD, (**C**) GSH-Px, (**E**) CAT, (**G**) MDA in liver and (**B**) SOD, (**D**) GSH-Px, (**F**) CAT, (**H**) MDA in kidney in STZ-treated mice. The values are reported as the mean ± SD of ten mice per group: (a) *P* < 0.01 and (b) *P* < 0.05 compared with NC groups; (c) *P* < 0.01, and (d) *P* < 0.05 compared with the MC groups

As Fig. 2 displayed, significant reductions in SOD, GSH-Px and CAT activities, and significant elevations of MDA contents were observed in STZ-induced diabetic mice as compared to the NC group (*P* < 0.01), respectively. In this work, the SOD and GSH-Px activities of En-MZPS in liver were expressed dose-dependently (Figs. 2A and B), however, the CAT activities of En-MZPS were not very dose dependent, at the tested doses of 800, 400, and 200 mg/Kg (Fig. 2C), respectively. The SOD and GSH-Px activities reached the maximum of 173.2 ± 14.38 and 93.9 ± 11.24 U/mg prot in the high dosage of En-MZPS, which were significantly higher than that in MC group (74 ± 5.56 and 32 ± 5.40 U/mg prot, both *P* < 0.01). As for CAT activities, it reached 245.8 ± 19.26 U/mg prot in the low dosage of En-MZPS, which was higher than that in diabetic control group (97.5 ± 6.89 U/mg prot) (Fig. 2C). Nevertheless, inverse tendency of En-MZPS on SOD, GSH-Px, and CAT activities in kidney were depicted in Figs. 2E, F, and G. As shown in Fig. 2E, significant attenuations in SOD activities were observed after the administration of Ac-, Al- and En-MZPS at three doses of 800, 400, 200 mg/Kg (41.9 ± 2.12, 61.3 ± 7.57, 85.1 ± 3.97, 71.3 ± 8.58, 89.0 ± 9.13, 64.5 ± 12.40, 65.1 ± 4.98, 69.2 ± 3.85 and 76.1 ± 7.55 U/mg prot, respectively), when compared to diabetic control group (27.4 ± 8.03 U/mg prot). Similar results were observed in GSH-Px activities (131.1 ± 9.17, 102.7 ± 4.87, 97.5 ± 9.13, 106.9 ± 7.83, 108.5 ± 6.01, 145.3 ± 8.64, 145.6 ± 3.26 and 152.4 ± 4.88 U/mg prot, respectively) (Fig. 2F), and CAT activities (66.1 ± 7.10, 99.4 ± 5.34, 162.8 ± 126.62, 90.3 ± 2.84, 111.5 ± 10.00, 128.0 ± 7.62, 130.4 ± 6.69, 162.8 ± 13.41, and 170.1 ± 15.26 U/mg prot, respectively) (Fig. 2G). Glibenclamide-treated mice also manifested significant fall of the SOD, GSH-Px, and CAT activities in liver and kidney compared to STZ-treated mice.

As shown in Figs. 2D and H, the contents of MDA were significantly increased (*P* < 0.01) in liver and kidney of diabetic mice when compared to NC group. Obviously, we could conclude that En-MZPS had strong potential antioxidant effects at high dose (800 mg/Kg) in liver (Fig. 2D), whereas at low dose (200 mg/Kg) in kidney (Fig. 2H).

### The assays of serum biochemistry

**Fig. 3:**
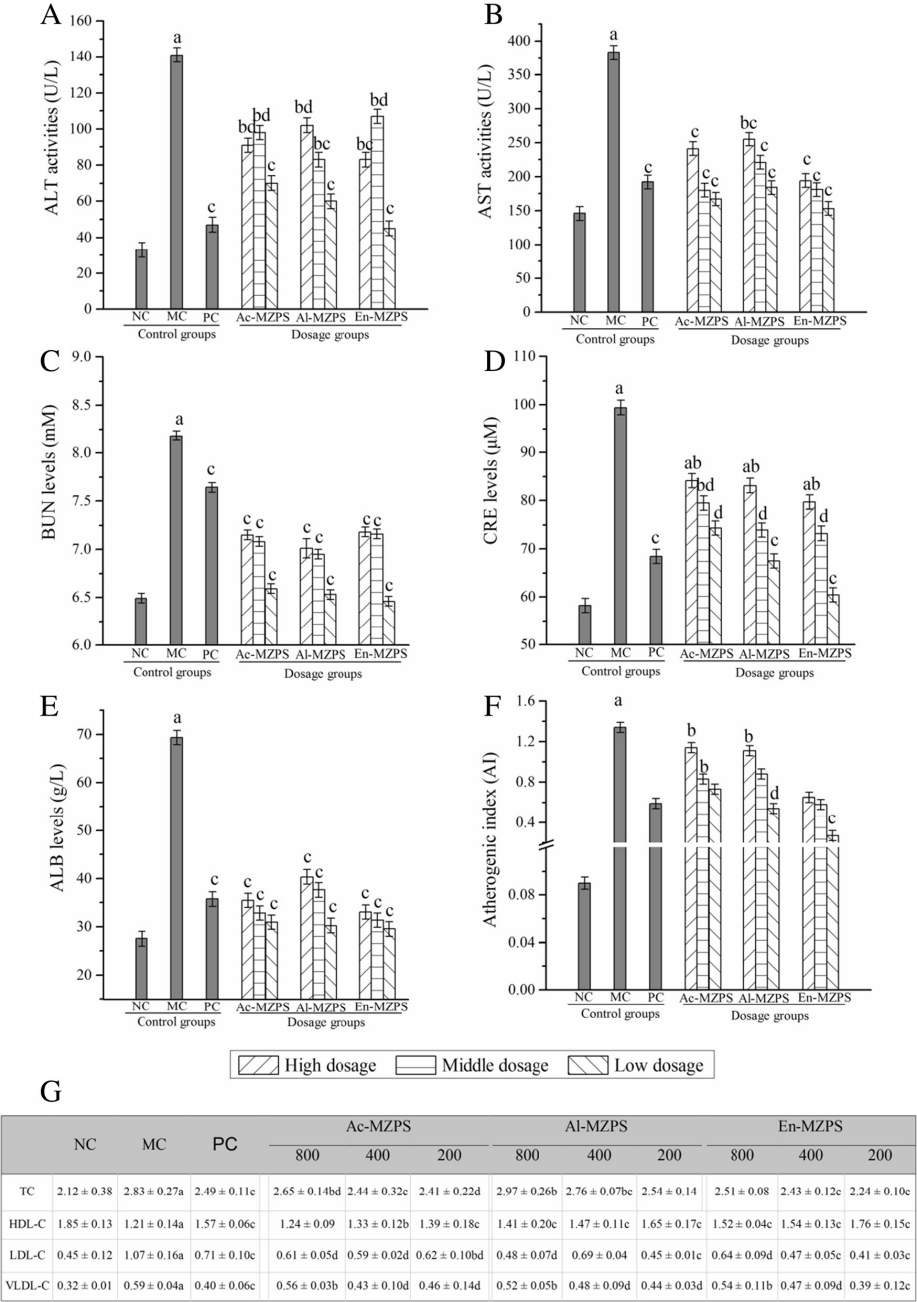
Effects of Ac-, Al, and En-MZPS on (**A**) ALT activities, (**B**) AST activities, (**C**) BUN levels, (**D**) CRE levels, (**E**) ALB levels, (**F**) AI levels and (**G**) levels of TC, HDL-C, LDL-C, and VLDL-C in STZ-treated mice. The values are reported as the mean ± SD of ten mice per group: (a) *P* < 0.01 and (b) *P* < 0.05 compared with NC groups; (c) *P* < 0.01, and (d) *P* < 0.05 compared with the MC groups

Several enzymes and substances in serum were used as biochemical markers for early liver and kidney damage, such as AST and ALT for liver, and BUN, CRE and ALB for kidney. As displayed in Fig. 3, mice treated with STZ alone showed liver damage and kidney damage as evidenced by significant increase in the serum activities of AST and ALT, and levels of BUN, CRE and ALB when compared with NC groups (all with *P* < 0.01). Interestingly, pretreatment with Ac-, Al- and En-MZPS significantly suppressed elevations in these indexes (*P* < 0.01 or *P* < 0.05) compared with MC groups, especially En-MZPS at the dose of 200 mg/Kg (*P* < 0.01) (Figs. 3A–E). The results signified that supplementation with Ac-, Al- and En-MZPS could depress the activities of AST and ALT, levels of BUN, CRE and ALB in STZ-intoxicated mice and appeared to be protective in undermining the deleterious effects of STZ in liver and kidney.

Figures 3F and G showed that the effects of three polysaccharides on AI, and serum TC, LDL-C and VLDL-C levels in the diabetic mice were significantly (*P* < 0.01 for TC, LDL-C, VLDL-C, and AI) higher, while the HDL-C levels were significantly (*P* < 0.01) lower than those in the MC groups. After 2 weeks of gavage administration, the contents of TC, LDL-C, and VLDL-C were decreased significantly and the contents of HDL-C were increased significantly in the three dose groups than those in the MC groups. Compared to the MC mice, the TC, LDL-C, VLDL-C, and AI levels in group of En-MZPS at 200 mg/Kg declined by 19.78 ± 1.42, 61.68 ± 1.33, 33.90 ± 5.47, and 79.85 ± 1.89 %, respectively. In the mean time, the HDL-C increased by 45.45 ± 2.34 % at the same dosage treatment.

### Histopathological study of liver and kidney cortex

**Fig. 4:**
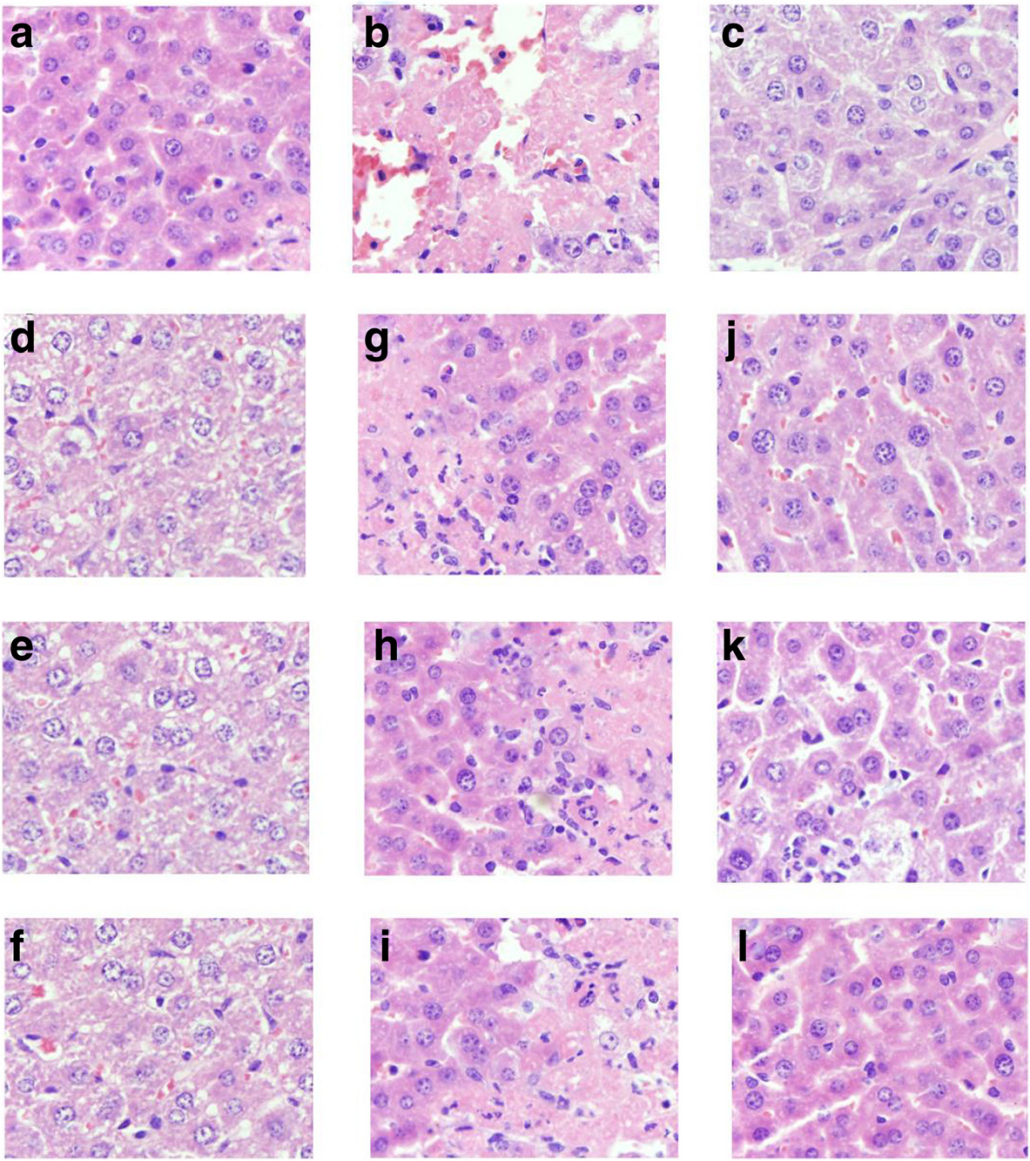
Representative photomicrographs of liver histopathology (400×). **a**: liver in mice of NC groups showing normal cellular architecture of hepatic tissue; (**b**): liver of mice in MC groups after injection of STZ (120 mg/Kg) showing cellular degeneration, hepatocyte necrosis, and lipid droplet accumulation; (**c**): liver of mice in PC groups; (**d**-**f**): liver of mice fed with Ac-, Al-, and En-MZPS at dosage of 800 mg/Kg showing mild architectural damage; (**h-i**): liver of mice fed with Ac-, Al-, and En-MZPS at dosage of 400 mg/Kg showing mild architectural damage with few showing abnormal structure; (**j-l**): liver of mice fed with Ac-, Al-, and En-MZPS at dosage of 200 mg/Kg showing almost normal histology similar to control mice

**Fig. 5:**
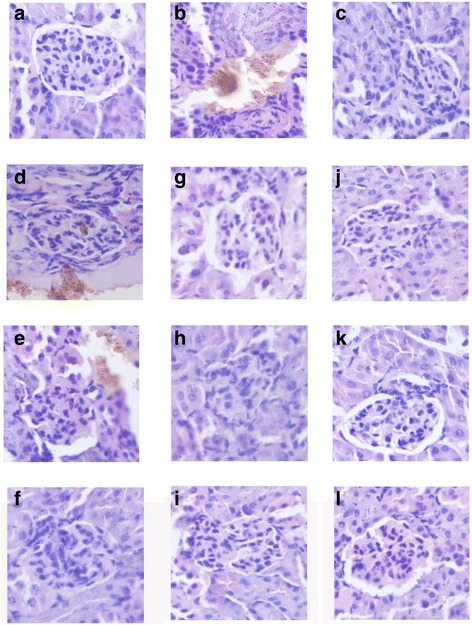
Representative photomicrographs of kidney cortex histopathology (400×). **a**: kidney cortex of mice in NC groups showing normal architecture; (**b**): kidney cortex of mice in MC groups after injection of STZ (120 mg/Kg) showing complete destruction of normal architecture of kidney cortex including shrunken proximal renal tubular cells with chromatin condensation, cytoplasm with vacuolation and cells with swelling and lysis; (**c**): kidney cortex of mice in PC groups; (**d-f**): kidney cortex of mice fed with Ac-, Al-, and En-MZPS at dosage of 800 mg/Kg showing mild architectural damage; (**g-i**): kidney cortex of mice fed with Ac-, Al-, and En-MZPS at dosage of 400 mg/Kg showing mild architectural damage with few showing abnormal structure; (**j-l**): kidney cortex of mice fed with Ac-, Al-, and En-MZPS at dosage of 200 mg/Kg showing almost normal histology similar to control mice

In the current study, histopathological observation of the liver and kidney cortex were performed to corroborate the evidence from biochemical analyses (Figs. 4 and 5).

As shown in Fig. 4, in comparison with the normal cellular architecture of hepatic tissue of mice in the NC group (Fig. 4a), extensive liver damage in diabetic mice were observed after the injection of STZ characterized by cellular degeneration, hepatocyte necrosis, and lipid droplet accumulation (Fig. 4b). The pretreatment with Ac-, Al- and En-MZPS exhibited a marked improvement in the liver histopathology evidenced by a diminution of necrotic zones and attenuation of lipid droplet accumulation against STZ-induced histological alteration (Figs. 4d–l), especially En-MZPS at 200 mg/Kg, suggesting that administration of low dosage of En-MZPS could achieve almost complete normalization of the liver tissues (Fig. 4l).

Figure 5 showed kidney cortex histology under light microscopy. Renal tubules in mice of MC group showed loss of brush border and integrity, vacuolation of tubular epithelial cells and acute tubular necrosis after STZ injection (Fig. 5b) as compared to thee NC group with normal architecture (Fig. 5a). Interestingly, these pathological changes were markedly attenuated with the three tested polysaccharides (Figs. 5d–l), especially using En-MZPS at low dosage (200 mg/kg) (Fig. 5l).

### Acute toxicity study

Mice (both males and females) administered Ac-, Al-, and En-MZPS did not induce any clinical signs of toxicity either immediately or during the post-treatment period at dosage of 5000 mg/Kg, indicating that Ac-, Al-, and En-MZPS were all practically non-toxic substances [29].

## Discussion

Diabetes mellitus (DM), characterized by hyperglycemia and abnormalities in carbohydrate, lipid and protein metabolism, was one of the most common human metabolic diseases. And The STZ-induced diabetic animal is thus considered as an animal model of type I diabetes and hyperlipidemia [30]. The precise working mechanisms about the anti-diabetic effects were still not fully understood up till now [31, 32]. However, accumulating studies had suggested that oxidative stress played a crucial role in the pathogenesis and progression of diabetes and its complications [33]. Baynes et al. proved that uncontrolled ROS production often led to damage in cellular macromolecules (DNA, lipids and proteins), contributing to the progress of diabetic complications and different organ damage [12]. The high toxicity of STZ engendered lipid peroxidation and depletion of the antioxidant enzymes and eventually liver damage. The present study reported the antioxidative activities of three extractable mycelium zinc polysaccharides (Ac-, Al- and En-MZPS) from *P. djamor* on liver and kidney of normal and STZ-induced diabetic mice using enzymatic and non-enzymatic methods under *in vivo* conditions.

Liver and kidney, which were the sensitive organs with chemotherapy cytotoxicity, played important roles in glucose metabolism of diabetic animals [34]. The AST and ALT activities in serum had been used as biochemical markers for liver damage [35], and the enzyme activities were markedly heightened when liver damaged occurred [36]. It had been reported that the leakage of large quantities of enzymes into the bloodstream was associated with massive centrilobular necrosis, ballooning degeneration, and cellular infiltration of the liver [37, 38]. This was consistent with our results presented in Figs. 3A, B and 4b. The other two parameters including BUN and CRE levels had been widely used in clinical to reflect the physical status of kidney [39]. The BUN, first endogenous substance produced by the decomposition of proteins in liver, was excreted by the filtration of glomerulus. The CRE, a byproduct of creatine and phosphocreatine catabolism, was endogenously produced and released into body fluids. And its clearance measured as an indicator of glomerular filtration rate [40]. The suppression of AST and ALT activities, BUN and CRE levels, as well as ALB levels in serum brought about by Ac-, Al- and En-MZPS indicated the stabilization of plasma membrane as well as repair of hepatic and nephritic tissue damages caused by STZ.

In addition, in order to understand the causal roles of the antioxidant activities and protective effects of the three polysaccharides on liver and kidney damage induced by STZ, the activities of antioxidant enzymes (SOD, GSH-Px and CAT) and the lipid contents (MDA) in the liver and kidney homogenate were determined. Antioxidant enzymes, converted active oxygen molecules into non-toxic compounds, formed the first line of defense against ROS in the organism during oxidative stress [24]. Free radical scavenging enzymes including SOD, GSH-Px and CAT were the first line of defense against oxidative injury in mammalian systems [41]. The role of SOD was to catalyze the superoxide anion into hydrogen peroxide and oxygen and reduced the intracellular concentration of superoxide [42]; CAT hold the function that catalyzed the decomposition of hydrogen peroxide into less-reactive gaseous oxygen and water molecules [43]; The biochemical function of GSH-Px was to catalyze lipid hydroperoxides to their corresponding alcohols and to catalyze free hydrogen peroxide to H_2_O [44], resulting in the prevention of ROS formation [35, 45, 46]. Therefore, the statuses of these antioxidant enzymes were appropriate indirect ways to assess the pro-oxidant-antioxidant status in tissues [47]. In addition, the ROS could interact with polyunsaturated fatty acids, leading the formation of lipid peroxidation (MDA), which was considered to be a hallmark of oxidative stress causing tissue damages [45]. Lipid peroxide was a primary parameter which could be considered as a marker of oxidative injury *in vivo* [48]. Totally, in our study, STZ injection caused the decrease in SOD, GSH-Px, and CAT activities, and the increase in MDA contents of mice as compared with the NC groups, suggesting that oxidative damage occurred in the liver and kidney. And the pretreatment with three polysaccharides significantly reversed these deleterious changes. These effects might, at least in part, be derived from the capability of MZPS to scavenge ROS.

Oral administration of polysaccharides from *P. djamor* caused significantly lower GLU levels in STZ-induced diabetic mice, similar to the findings of other fungal polysaccharides [18, 20, 37]. Moreover, MZPS could improve the loss of body weight in STZ-induced diabetic mice, which may be attributed to the ability of MZPS to significantly improve glucose homeostasis. In addition, elevated TC, LDL-C, VLDL-C, and low HDL-C levels were well established risk factors for coronary heart disease [49]. In this regard, MZPS administrations markedly lowered TC, LDL-C, and VLDL-C levels, and elevated the HDL-C levels in diabetic mice, suggesting that MZPS had potential effects in improving hyperglycemia and hyperlipemia in STZ-induced diabetic mice.

Altogether, these findings suggested that the MZPS, used in the present study, were novel bioactive compounds responsible for the anti-diabetic effects, and thus provided new insights into the anti-diabetic effects of polysaccharides from *P. djamor*, as well as the potential mechanisms. There were two possible reasons for hypoglycemic effects of MZPS deduced from present data. First, the MZPS could act as free radical scavengers to quench ROS, possessing a direct anti-oxidative activity. Alternatively, MZPS could indirectly relieve the oxidative damage of liver and kidney by improving the antioxidant enzyme activities and reducing the lipid contents, *i.e.*, MZPS had potential effects in rescuing liver functions in stabilizing the gluconeogenesis in the liver, thus improving glucose metabolism in diabetic mice (Fig. 2). Second, MZPS showed the inhibitory effects on vascular inflammation, reflected by TC, HDL-C, LDL-C, VLDL-C and AI levels from Figs. 3F and G, possibly resulting in amelioration of inflammatory damage in STZ-targeted tissues.

However, previous literatures had reported the potential relationships between lipid metabolism difference and bacterial community change, indicating that the colonic microflora played important biochemical activities in regulating serum lipids and cholesterol by taking part in metabolism [50]. Lots of intestinal bacteria, including *Bacteroides sp.*, *Eubacterium sp.*, *Butyrivibrio proteoclasticus*, *Bifidobacterium bifidum*, *Lactobacillus fermentum*, and *Lactobacillus reuteri*, had been found increased in mice colon after the intake of polysaccharides, suggesting the potential effects of polysaccharides on the mice lipid metabolism [51]. So, desiderated attentions should be paid on the changed colon bacteria after the treatment of these three polysaccharides, and there is a need to continue to explore the relationship between free radicals, diabetes, and its complications. And more studies are needed to elucidate the mechanisms of action and the efficacy of these three extractable zinc polysaccharides administrations as an adjuvant therapy for diabetic patients in an effort to expand treatment options.

## Conclusions

From the results, we concluded that MZPS exerted protective effects in experimental diabetic mice induced by STZ injection, possibly by reducing oxidative stress of liver and kidney, and hence, we asserted that MZPS had potential effects in reducing the development of diabetic complications. Above all, the results of this study may provide a mechanistic basis for MZPS using as a potentially natural and functional food for the prevention and alleviation of diabetes and its complications.

## Abbreviations

Ac-MZPS: acidic-extractable mycelium zinc polysaccharides
AI: atherogenic index
ALB: albumin
Al-MZPS: alkalic-extractable mycelium zinc polysaccharides
ALT: alamine aminotransferase
AST: aspertate aminotransferase
BUN: urea nitrogen
CAT: catalase
CRE: creatinine
DM: diabetes mellitus
En-MZPS: enzymatic-extractable mycelium zinc polysaccharides
GLU: blood glucose
GSH-Px: GSH peroxide
HDL-C: high-density lipoprotein cholesterol
IDF: international diabetes federation
LDL-C: low-density lipoprotein cholesterol
MDA: malondialdehyde
MZPS: mycelium zinc polysaccharides
ROS: reactive oxygen species
SOD: superoxide dismutase
STZ: streptozotocin
T-AOC: total antioxidant capacity
TC: total cholesterol
VLDL-C: very low-density lipoprotein cholesterol
